# Study of the Seasonal Variations of the Fatty Acid Profiles of Selected Macroalgae

**DOI:** 10.3390/molecules26195807

**Published:** 2021-09-25

**Authors:** Tatiana Pereira, André Horta, Sónia Barroso, Susana Mendes, Maria M. Gil

**Affiliations:** 1 MARE—Marine and Environmental Sciences Centre, Polytechnic of Leiria, Cetemares, 2520-620 Peniche, Portugal; tatiana.m.pereira@ipleiria.pt (T.P.); andre.horta@ipleiria.pt (A.H.); sonia.barroso@ipleiria.pt (S.B.); 2 Division of Aquaculture, Upgrading and Bioprospection, Portuguese Institute for the Sea and Atmosphere (IPMA), Avenida Alfredo Magalhães Ramalho, 6, 1495-165 Algés, Portugal; 3 Research Institute for Medicines, Faculty of Pharmacy, Universidade de Lisboa, Av. Professor Gama Pinto, 1649-003 Lisboa, Portugal; 4 MARE—Marine and Environmental Sciences Centre, ESTM, Polytechnic of Leiria, Cetemares, 2520-620 Peniche, Portugal; susana.mendes@ipleiria.pt

**Keywords:** fatty acids, seasonality, *Fucus* *spiralis*, *Bifurcaria* *bifurcata*, *Ulva* *lactuca*, *Saccorhiza* *polyschides*

## Abstract

Due to the high consumption of fat-rich processed foods, efforts are being done to reduce their saturated fat (SFA) contents and replace it with polyunsaturated fatty acids (PUFA), creating a necessity to find alternative PUFA sources. Macroalgae, being a promising natural source of healthy food, may be such an alternative. The fatty acid (FA) profile of *Fucus spiralis*, *Bifurcaria bifurcata*, *Ulva lactuca*, and *Saccorhiza polyschides* were determined through direct transesterification and their seasonal variation was studied. *F. spiralis* showed the highest FA content overall, *B. bifurcata* presented the higher PUFA amounts, and *U. lactuca* and *S. polyschides* the higher SFA. The production of FA was shown to be influenced by the seasons. Spring and summer seemed to induce the FA production in *F. spiralis* and *B. bifurcata* while in *U. lactuca* the same was verified in winter. *U. lactuca* presented a ω6/ω3 ratio between 0.59 and 1.38 while *B. bifurcata* presented a ratio around 1.31. The study on the seasonal variations of the macroalgal FA profile can be helpful to understand the best season to yield FA of interest, such as ALA, EPA, and DHA. It may also provide valuable information on the best culturing conditions for the production of desired FAs.

## 1. Introduction

Due to its convenience, the consumption of highly processed foods is increasing each year. However, this habit has become costly for the health of its consumers and a fat-rich diet has been associated with the rise of various non-communicable diseases [1,2]. The high amounts of fatty acids (FA) present in these types of foods, especially saturated fatty acids (SFA), have been associated with the increase of LDL (low-density lipoprotein) cholesterol and the incidence of diseases such as cardiovascular diseases and diabetes [3,4]. For this reason, efforts are being made to reduce the SFA contents in foods and replacing them with unsaturated fatty acids [2,4].

PUFA, such as ω3 and ω6, are essential for the human diet and have been associated with the reduction of cholesterol, regulation of blood pressure, and in decreasing the risk of developing diabetes and cardiovascular diseases [3,5,6]. However, excessive consumption of ω6 in detriment of ω3 is being reported to contribute to the incidences of obesity, diabetes, and atherosclerosis [7]. Thus, a balance between the two is highly recommended to achieve the benefits and reduce the possible harmful effects.

Traditionally, PUFA were obtained by the consumption of oily fish (salmon, tuna, cod) and their fish oil extracts [8]. However, due to climate changes, overfishing, and intensive aquaculture, these kinds of fish have seen a decrease in their PUFA contents over time, and their ability to satisfy the growing demand is being questioned [8,9]. Because of that, there is a need to find novel and alternative sources of PUFA. Seeing that the fish get their PUFA from their algae-based food, it is possible to obtain the desired compounds directly from their original sources [8,10,11].

Algae are well known to be a source of several bioactive nutritional compounds indispensable for a healthy diet. From those, marine algae are reported to present high quantities of PUFA despite their low quantities of lipids [12,13,14]. Seeing as algae can produce long-chain fatty acids (LC-FA) such as docosahexaenoic acid (DHA) and eicosapentaenoic acid (EPA), their biochemical composition is being investigated to better understand their potential applications [8]. Nevertheless, the production of algal contents varies greatly in response to several factors, namely geographical location, seasonality, internal distribution, life cycle, and environmental factors [12,15,16]. Therefore, the study of the seasonal variations on the FA production in the different macroalgae can be helpful to understand the tendency of production of FA of interest, namely ALA (α-linolenic acid), EPA, and DHA, and the best harvest season to yield the best FA profile with the most nutritional potential. Regarding a potential industrial adoption of FA extractions using these macroalgae, the study of the most favorable seasons for the production of the FA of interest would indicate the best culturing conditions in which to stimulate the algae production of those FA. Taking this into consideration, the main objective of this study was the quantification of the fatty acid profile of four macroalgae, *Ulva lactuca*, *Bifurcaria bifurcata*, *Fucus spiralis*, and *Saccorhiza polyschides* collected from the coast of Peniche, Portugal, throughout the year and evaluating the seasonal variations in the FA profile of the macroalgae.

## 2. Results and Discussion

**Figure 1:**
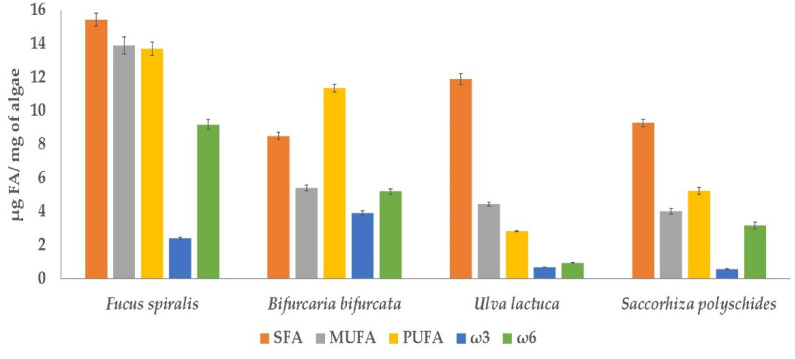
Comparison of the sums of saturated fatty acids (SFA), monounsaturated fatty acids (MUFA), polyunsaturated fatty acids (PUFA), omega-3 (ω3), and omega-6 (ω6) of *Fucus spiralis*, *Bifurcaria bifurcata*, *Ulva lactuca,* and *Saccorhiza polyschides* for samples collected in summer, presented as µg of fatty acid/mg of macroalgae. Values are expressed as mean ± SD (n = 3).

In this study, the quantification of fatty acids (FA) present in macroalgae readily available on the coast of Peniche was carried out. The screening was done on *Bifurcaria bifurcata*, *Fucus spiralis*, *Saccorhiza polyschides* (Ochrophyta), and *Ulva lactuca* (Chlorophyta). The different macroalgae presented distinct FA profiles in the four different seasons (winter, summer, spring, and autumn) (Table 1). In the case of *S. polyschides*, only the FA profile from summer was studied due to lack of biomass in the other seasons. Figure 1 displays the comparison of the sums of saturated fatty acids (SFA), monounsaturated fatty acids (MUFA), polyunsaturated fatty acids (PUFA), omega-3 (ω3), omega-6 (ω6) and the ω6/ω3 and PUFA/SFA ratios of the samples collected in summer.

### 2.1. FA Content in the Macroalgae

#### 2.1.1. *Fucus spiralis*

*Fucus spiralis*, when compared to the remaining macroalgae, was the one that presented the highest amount of the different FAs overall. In the samples of *F. spiralis*, it was possible to identify 28 different FAs, from C4 to C24. The macroalgae exhibited higher cumulative contents of SFAs, followed by PUFAs and lastly, MUFAs. Oleic (C18:1-ω9), palmitic (C16:0), arachidonic (AA, C20:4-ω6), and myristic (C14:0) acids were the most significant, presenting the highest concentrations, at 10.98 ± 0.52, 6.41 ± 0.33, 5.94 ± 0.28, and 4.01 ± 0.25 µg/mg, respectively (Table 1). Linolenic (LA, C18:2-ω6), arachidic (C20:0), stearidonic (C18:4), and eicosapentaenoic (EPA, C20:5-ω3) acids were identified in substantial quantities, ranging between 2.46 ± 0.15 and 1.89 ± 0.09 µg/mg (Table 1). There were also some amounts of stearic (C18:0, 0.95 ± 0.02 µg/mg), eicosatetraenoic (C20:4-ω3, 0.88 ± 0.02 µg/mg), palmitoleic (C16:1, 0.87 ± 0.04 µg/mg), and butyric (C4:0, 0.58 ± 0.02 µg/mg) acids (Table 1). All other FA identified presented quantities lower than 0.53 µg/mg (Table 1). No -linolenic acid (ALA) and docosahexaenoic acid (DHA) were identified for this species.

The FA profile of *F. spiralis* was overall in agreement with other published works, with the predominance of the oleic, palmitic, myristic, and arachidonic acids having been previously reported [16,17]. Most studies present higher contents of SFA [12,18,19,20,21] with only one reporting higher PUFA in samples from S. Miguel (Azores, Portugal) [22]. In the present study, a slightly higher sum of SFA was observed, closely followed by the sum of the MUFA and PUFA. The SFA content was mainly due to the abundance of palmitic acid, while the MUFA was caused primarily by the oleic acid and the PUFA by the AA.

As for the ω6/ω3 ratio, higher amounts of ω6 were verified, between 6.56 ± 0.68 and 9.18 ± 0.30 µg/mg, which was mainly caused by the high amounts of AA and LA (Table 1). The sum of ω3 ranged from 2.24 ± 0.23 to 2.77 ± 0.09 µg/mg and was only represented by the eicosatetraenoic acid and EPA (Table 1). Along with the ω6/ω3 ratio, the hypocholesterolemic/hypercholesterolemic (h/H) ratio is another important ratio in evaluating the nutritional value of the macroalgae, considering their FA profile and the known effects of the FA on the metabolism of the cholesterol [12,23,24]. This ratio is calculated by dividing the sum of the MUFA and the PUFA by the sum of the myristic (C14:0) and palmitic acids (C16:0). A h/H ratio of 2.54 to 2.78 was quantified (Table 1) and suggested a high nutritional value of the *F. spiralis* taking into consideration its FA composition.

Previous studies on *F. spiralis* from the coast of Portugal reported higher ω3 contents, resulting in lower ω6/ω3 ratios (0.84) [18]. Nevertheless, the majority of the studies seemed to point to the prevalence of ω6 over ω3 on this macroalgae species. In a different study on samples collected in Peniche (Portugal), higher ω6 contents were reported, at 22.46% of the total FA, against only 15.07% of total FA represented by ω3, resulting in a ω3/ω6 ratio of 0.67 [17]. This higher ω6 trend was also observed in samples harvested in S. Miguel (Azores, Portugal), Praia do Norte (Portugal), and on the Atlantic Coast of Morocco, which had ω6/ω3 ratios of 1.78, 2.09, and 2.88, respectively [19,20,22].

Seasonal variations and the macroalgae life cycle can influence the ω6/ω3 ratios, as evaluated in previous articles. Samples from S. Miguel and S. Maria, Azores, presented a ω6/ω3 ratio between 1.51 (winter, S. Maria) and 2.94 (summer, S. Miguel) and a h/H ratio ranging from 1.59 (summer, S. Maria) to 2.37 (summer, S. Miguel) [12]. Meanwhile, samples from S. Miguel from the juvenile phase of the life cycle presented a ω6/ω3 ratio of 2.07 while samples from the mature phase presented ratios of 2.67 [16]. The effect of the seasonal variations on the FA profile will be further addressed in Section 2.2.

#### 2.1.2. *Bifurcaria bifurcata*

*Bifurcaria bifurcata* presented the most variety of fatty acids, with 36 different FAs identified, from C4 to C24, and the highest concentration of PUFAs. The higher amounts were found for palmitic (3.93 ± 0.21 µg/mg), arachidonic (3.10 ± 0.15 µg/mg), and oleic (2.40 ± 0.14 µg/mg) acids (Table 1). Present in significant contents were the stearidonic (1.38 ± 0.04 µg/mg), α-linolenic (ALA, C18:3-ω3, 1.26 ± 0.10 µg/mg), and myristic (1.06 ± 0.03 µg/mg) acids (Table 1). These were followed by the palmitoleic (0.89 ± 0.05 µg/mg), eicosatetraenoic (0.74 ± 0.05 µg/mg), behenic (C22:0, 0.69 ± 0.04 µg/mg), and caproic (C6:0, 0.72 ± 0.04 µg/mg) acids (Table 1). EPA (0.86 ± 0.05 µg/mg) and LA (0.68 ± 0.03 µg/mg) were identified in low amounts and only traces of DHA (0.18 ± 0.01 µg/mg) were observed (Table 1). The remaining identified FA were found to have amounts less than 0.57 µg/mg (Table 1).

The resulting *B. bifurcata* FA profile was in agreement with the ones obtained by previous studies, with a predominance of the palmitic, arachidonic, and oleic acids [25,26,27,28,29]. However, the literature presents differences in the nature of the quantified FA, with some of the studies reporting higher concentrations of SFA [25,28,29] and others higher concentrations of PUFA [26,27]. These discrepancies could be the result of the distinct sample harvest locations and harvest seasons. Higher amounts of PUFA were found in samples collected during the summer months [26,27], while higher concentrations of SFA were found in samples from spring and autumn [25,29]. In the present study, higher amounts of PUFA were consistently observed in all seasons (Table 1).

At present, *Bifurcaria bifurcata* samples presented a ω6/ω3 ratio between 1.28 and 1.33 (Table 1). The ω3 content, which ranged between 3.16 ± 0.05 and 3.91 ± 0.15 µg/mg, was mainly due to the amounts of α-linolenic and eicosatetraenoic acids (Table 1). Higher amounts of ω6 were quantified (4.20 ± 0.11 and 5.21 ± 0.16 µg/mg) and were mostly represented by the AA (Table 1). The quantification of the h/H ratio resulted in a value around 3.37–3.45 (Table 1), showing great promise for the use of *B. bifurcata* to supplement a healthy diet due to its high nutritional value.

Similar ω6/ω3 ratios were reported in previous studies. A study with samples collected from Peniche (Portugal) presented a ω6/ω3 ratio of 1.73, while samples from Praia da Aguda (Portugal) and Camariñas (Spain) showed ratios of 1.22 and 1.41, respectively [26,27,29]. Lower ratios were obtained in samples from Ria de Aveiro (Portugal), at 0.46, and higher ratios were obtained for samples from the Atlantic Coast of Morocco, at 4.51 [25,28].

#### 2.1.3. *Saccorhiza polyschides*

Unlike the other Ochrophyta studied, *Saccorhiza polyschides* presented higher SFA content. In the samples, 26 distinct FAs from C4 to C22 were identified. Similarly to *B. bifurcata*, *S. polyschides* presented high amounts of palmitic (3.63 ± 0.19 µg/mg), arachidonic (2.07 ± 0.19 µg/mg), oleic (1.79 ± 0.16 µg/mg), and stearidonic (1.21 ± 0.04 µg/mg) acids (Table 1). There were also considerable amounts of the MUFA palmitoleic acid at 0.91 ± 0.05 µg/mg and the SFAs myristic, arachidic, butyric (C4:0), and caprylic (C8:0) acids ranging between 0.89 ± 0.04 and 0.63 ± 0.01 µg/mg (Table 1). LA (0.61 ± 0.05 µg/mg), *cis-*vaccenic (C18:1-ω7, 0.57 ± 0.03 µg/mg), lauric (0.53 ± 0.01 µg/mg), caproic (0.51 ± 0.01 µg/mg), and stearic (0.51 ± 0.01 µg/mg) acids were also identified (Table 1). EPA was also found at 0.41 ± 0.02 µg/mg, and the remaining FA identified presented contents below this value (Table 1). This species did not present any amounts of ALA or DHA.

In *S. polyschides*, only samples from summer were collected and the resulting profile was similar to the ones previously reported for this species, with greater quantification of palmitic acid, accounting for the overall higher prevalence of SFA, AA, and oleic acids [15,19,25,30]. Unlike most of the previous studies, a great amount of stearidonic acid was also identified (1.21 ± 0.04 µg/mg of macroalgae) (Table 1). Stearidonic acid was only previously quantified in samples from Galway, Ireland and in processed seaweeds from Ría de Arousa, Spain [31,32].

Its ω3 contents were lower than in the other brown macroalgae studied, at only 0.57 ± 0.02 µg/mg and predominantly represented by EPA (0.41 ± 0.02 µg/mg), and a ω6 content of 3.18 ± 0.20 µg/mg, primarily made up by AA (Table 1). The resulting ω6/ω3 ratio was of 5.60; nevertheless, it presented a h/H ratio of 2.05, proving its potential nutritional value (Table 1).

Previous studies on *S. polyschides* collected from the Peniche coast and from Buarcos reported ratios of 0.58 and 1.63, values much lower than the one obtained in the present study [18,30]. Different FA quantification methodologies and the changing environmental conditions in which the samples were exposed could have played a role in the different results. Nevertheless, similar ratios to those reported in the present study were reported for this macroalga from samples collected from the Coast of Morocco, Praia do Norte (Portugal), and Gulf of Cádiz (Spain), with ratios around 3.11, 6.62, and 10.9, respectively [19,25,33].

As previously mentioned, the distinct harvest seasons could have contributed to the distinct results. The seasonal variation of this macroalgae was not able to be determined due to insufficient biomass, however, a previous study on this variation performed by Barbosa et al. [15] showed that the production of total fatty acids in *S. polyschides* was promoted by the colder months and a tendency to decrease the production of MUFA and PUFA in warmer months. Nevertheless, it is well known that the algal components are dependent on a number of internal and external factors, namely geographical localizations and internal differentiation, and thus these results could not be accurately compared.

In another work, Schmid and Stengel [31] investigated the FA variation in the different structural components of macroalgae (holdfast, stipe, blade, and the tip of the blade), verifying that distinct parts present variations in the FA quantification. It was shown that in terms of PUFA, there were higher amounts on the tip of the blades as opposed to the other locations, while SFA and MUFA were more prevalent in the holdfast [31]. The ω3/ω6 ratio also varied internally, with the tip of the blade presenting a ratio of 0.8 and the remaining structures presenting ratios around 0.4–0.3 [31].

#### 2.1.4. *Ulva lactuca*

*Ulva lactuca* is one of the most studied marine macroalgae, mainly due to ready availability caused by its widespread distribution [34,35].

In the present study, 32 distinct FAs ranging from C4 to C22 were identified. This macroalga presented much higher SFA contents (than MUFAs and PUFAs) of all the samples investigated. High amounts of palmitic acid (6.20 ± 0.32 µg/mg) followed by *cis-*vaccenic (C18:1-ω7, 2.49 ± 0.12 µg/mg), stearidonic (1.04 ± 0.02 µg/mg), palmitoleic (0.99 ± 0.03 µg/mg), and behenic (0.91 ± 0.03 µg/mg) acids were found, especially in the samples collected in winter (Table 1). ALA and LA were quantified at 0.74 ± 0.02 and 0.56 ± 0.01 µg/mg, respectively (Table 1). Butyric (C4:0, 0.87 ± 0.04 µg/mg), oleic (0.81 ± 0.02 µg/mg), capric (C10:0, 0.73 ± 0.01 µg/mg), lauric (C12:0, 0.70 ± 0.02 µg/mg), myristic (0.66 ± 0.00 µg/mg), caprylic (0.64 ± 0.02 µg/mg), and caproic (0.62 ± 0.01 µg/mg) acids were also present (Table 1). The remaining FA were found in amounts below 0.49 µg/mg (Table 1). No traces of EPA and DHA were found.

The resulting FA composition was overall in agreement with previous quantifications of the FA present in *U. lactuca*, with higher amounts of palmitic acid and *cis-*vaccenic acid [34,36]. High SFA contents were quantified, primarily because content of palmitic acid was 60–80% higher than that of the second most abundant FA, *cis-*vaccenic acid, especially when the samples were collected in winter (Table 1). Frequently, the studies do not identify C18:1 isomer, presenting it only as C18:1 or as C18:1n [11,37], a point that was addressed by McCauley et al. [35], who mentioned that the use of FAME standards without C18:1ω7 can induce the assignment of C18:1ω9 to the C18:1ω7 peak due to its close retention times.

The only Chlorophyta studied presented lower amounts of ω3 and ω6 when compared with the rest of the macroalgae. However, it was the only macroalgae to present similar amounts of ω3 and ω6. *U. lactuca* presented the biggest production of ω3 in winter, resulting in a ω6/ω3 ratio of only 0.59, due to a 41% higher production of ω3 than ω6 (Table 1). The ω6/ω3 ratio underwent variations throughout the seasons and reached the highest ratio in summer at 1.38, with a decrease of ω3 and increase of ω6 (Table 1). The quantification of ω3 ranged between 0.69 ± 0.01 and 1.16 ± 0.02 µg/mg and was predominantly composed by ALA, while the sum of ω6 was between only 0.69 ± 0.02 and 1.01 ± 0.01 µg/mg and represented mainly by LA (Table 1). Even though *Ulva lactuca* presented the healthiest ω6/ω3 ratio (in winter and autumn) of all the studied macroalgae, it also presented the lowest h/H ratio, at only around 1.17 and 1.41 (Table 1), which was the result of its high SFA concentration.

In the literature, samples of *U. lactuca* are most often reported to have ω6/ω3 ratios close to 1 [11,38,39,40]. Higher ratios of 2.97 and 4.1 were found in samples from Spain and the southeast coast of Sri Lanka [33,41]. In the present study, a ω6/ω3 ratio of 0.59 was obtained for winter samples, which is well below the ones most often reported for this macroalgae. Nevertheless, similar results were reported in comparison studies. A study that compared the harvest months (June and November) of *Ulva lactuca* collected from Galway Bay, Western Ireland showed that the samples from June presented a ω6/ω3 ratio of 0.6 while samples from November presented a ratio of 0.2 [36]. In another case, the comparison of the effect of cultivation conditions on FA production concluded that low nutrition levels increased the production of SFA and decrease the PUFA [35]. In terms of the ω6/ω3 ratio, in low-nutrient conditions there was a ratio of 0.74 while in the high-nutrient environment the ratio reached 0.23. This study showed that there is an increase in the production of ω3 in highly nutritious conditions, whereas in low-nutrient conditions there is an increase in ω6 along with a reduction of ω3 production [35].

#### 2.1.5. Comparison of the FA Profiles of the Different Macroalgae

The FA profiles of three Ochrophytas (*F. spiralis, B. bifurcata,* and *S. polyschides*) and one Chlorophyta (*U. lactuca*) were studied. Palmitic acid was the most predominant FA in *B. bifurcata*, *U. lactuca,* and *S. polyschides* while in *F. spiralis* it was oleic acid (Table 1). AA was the second most abundant FA in all Ochrophyta species while in the only Chlorophyta, the second most abundant was *cis-*vaccenic acid (Table 1).

Oleic acid is often obtained from sunflower and olive oils, which contain 83 and 71 g/100 g, respectively [42]. *F. spiralis* was shown to be a good source of oleic acid, due to its high production of this FA, especially during the summer, when it reaches 10.98 ± 0.52 µg/mg (Table 1). Lower contents were found in the remaining macroalgae, at only 2.40 ± 0.14, 1.79 ± 0.16, and 0.81 ± 0.02 µg/mg in *B. bifurcata*, *S. polyschides*, and *U. lactuca* (Table 1). The production of oleic acid presented statistical differences between all seasons with the exception of autumn/winter in *F. Spiralis* (ANOVA, Tukey, *p*-value < 0.05) and only in autumn/summer and autumn/spring in *U. lactuca* (Kruskal–Wallis, Games-Howell, *p*-value < 0.05) (Table 1).

LA and ALA are essential FA and are precursors for the production of AA (ω6) and EPA and DHA (ω3), respectively [11]. High quantities of LA were found in *F. spiralis*, at 2.46 ± 0.15 µg/mg, while *B. bifurcata*, *S. polyschides*, and *U. lactuca* only presented maximums of 0.68 ± 0.03, 0.61 ± 0.05, and 0.56 ± 0.01 µg/mg, respectively (Table 1). The seasons seemed to significantly influence the amount of LA produced, with *F. spiralis* presenting differences in winter/spring and autumn/spring (Kruskal–Wallis, Games-Howell, *p*-value < 0.05), and *B. bifurcata* and *U. lactuca* presenting differences in all seasons except in autumn/winter and winter/summer, respectively (ANOVA, Tukey, *p*-value < 0.05) (Table 1).

Significant quantities of AA were found in all studied macroalgae species, except for *U. lactuca*, being verified at 5.94 ± 0.28 µg/mg in *F. spiralis,* 3.10 ± 0.15 µg/mg in *B. bifurcata,* and 2.07 ± 0.19 µg/mg in *S. polyschides* (Table 1). Statistically significant differences were seen in winter/summer and autumn/summer in *F. spiralis* (Kruskal–Wallis, Games-Howell, *p*-value < 0.05) and in all seasons with the exception of autumn/winter and spring/summer in *B. bifurcata* (ANOVA, Tukey, *p*-value < 0.05) (Table 1).

ALA was present in *B. bifurcata* and *U. lactuca*, with the former presenting almost double the ALA content, while in *F. spiralis* only low contents were found and in *S. polyschides* only traces (Table 1). There were differences in production of ALA in *U. lactuca* and *B. bifurcata*, with all seasons seeming to influence the FA content, with the exception of autumn/spring and autumn/winter in *B. bifurcata* (ANOVA, Tukey, *p*-value < 0.05) (Table 1).

As for the EPA and DHA, *U. lactuca, B. bifurcata,* and *S. polyschides* presented EPA and only *B. bifurcata* presented traces of DHA (Table 1). Only the concentration of EPA in *B. bifurcata* presented differences between seasons (ANOVA, Tukey, *p*-value < 0.05) (Table 1).

*F. spiralis* and *S. polyschides* presented greater amounts of ω6 than ω3, while *B. bifurcata* and *U. lactuca* exhibited ω6/ω3 ratios closer to 1 (Table 1). *U. lactuca* was the only one to yield ratios as low as 0.59 (Table 1). As previously mentioned, a balance between ω3 and ω6 is needed to achieve its health benefits, and thus it seems that only *U. lactuca* and *B. bifurcata* exhibited “healthier” ratios.

*U. lactuca* presented a good ratio well below 1 in winter; however, its overall ω3 production was lower than that verified in the previously mentioned macroalgae, reaching its higher contents at 1.16 ± 0.02 µg/mg in addition to presenting high SFA contents that could counterbalance its potential benefits (Table 1).

The studied macroalgae presented high h/H ratios, with *B. bifurcata* presenting the highest at 3.45, followed by *F. spiralis* (2.78), *S. polyschides* (2.05), and *U. lactuca* (1.41) (Table 1).

### 2.2. Seasonal Influence on the Production of FA

It is known that macroalgae produce different components in response to outside factors. One of those factors is the environmental conditions to which the macroalgae are exposed. In this study, as it was expected, the different seasons seemed to influence the macroalgal FA content. In the case of *S. polyschides*, only samples from summer were collected, so this macroalga will not be discussed in this section, and only *F. spiralis*, *B. bifurcata,* and *U. lactuca* will be further explored.

*F. spiralis* presented higher SFA and PUFA concentrations in samples from spring followed by summer, with a decrease of 4–21% in its abundance when compared with the rest of the seasons. The MUFA showed higher variations throughout the seasons, being most abundant in summer at quantities 15–43% higher than the other seasons.

Regarding *B. bifurcata*, the seasonal changes in the SFA, MUFA, and PUFA were less accentuated. Higher contents were obtained from samples collected in summer, at 8.51 ± 0.22 (SFA), 5.42 ± 0.17 (MUFA), and 11.36 ± 0.23 (PUFA) µg/mg (Table 1), representing only an increase of 8–18% compared to samples from the least productive season, autumn.

The season which yielded higher contents of SFA, MUFA, and PUFA in *U. lactuca* was winter. In SFA the seasonal variations were of only 8–12%, while bigger changes were verified in the MUFA (15–28%) and PUFA (14–21%) contents when compared to the results from winter and the remaining seasons.

Specific seasons seemed to induce the production of specific FA, being verified dramatic variations in the amount found at different times of the year. Some examples of these variations were the prevalence of oleic acid in *F. spiralis,* which was more emphatic in summer and spring (10.98 ± 0.52 and 8.71 ± 0.75 µg/mg; Table 1) with an increase of about 20–54% in summer and 40–42% in spring when compared with the other seasons. The same was verified in *B. bifurcata* but with a more modest increase of only 5–15% in summer and 5–11% in spring. Only in *F. spiralis* there were found statistically significant differences, with all seasons (except autumn/winter) seeming to influence the production of oleic acid (ANOVA, Tukey, *p*-value < 0.05) (Table 1). With EPA the differences reached up to 43% in *B. bifurcata* (*p*-value > 0.05) and 26% in *F. spiralis* between spring and autumn (*p*-value > 0.05) (Table 1). LA contents varied by 26% in *B. bifurcata* and 31% in *F. spiralis,* when comparing the most productive seasons, summer and spring, respectively, to the least productive season, autumn. *B. bifurcata* presented statistically significant differences in production of LA between all seasons except autumn/winter (ANOVA, Tukey, *p*-value < 0.05), while *F. spiralis*, only presented differences in winter/spring and autumn/spring (Kruskal–Wallis, Games-Howell, *p*-value < 0.05) (Table 1).

In *U. lactuca* the most drastic variations were verified in the production of *cis-*vaccenic and ALA, with the samples from winter presenting increases of about 54%, 49%, and 23% in the production of *cis-*vaccenic and 54%, 36%, and 42% in the ALA content when compared to summer, spring, and autumn, respectively. Differences were verified in the *cis-*vaccenic and ALA production in all seasons, except in the production of *cis-*vaccenic in spring/summer (ANOVA, Tukey, *p*-value < 0.05) (Table 1).

The production of ω3 and ω6 also seemed to present seasonal variations in all macroalgae. *F. spiralis* yielded higher amounts of ω3 in spring and lower amounts in summer, accounting for a decrease of 19%. Greater amounts of ω6 were verified in spring and summer (9.18 ± 0.30 and 8.87 ± 0.38 µg/mg, respectively; Table 1) and lower in winter and autumn (6.60 ± 0.04 and 6.56 ± 0.68 µg/mg, respectively; Table 1), which represented a reduction around 26–29% throughout the year.

In *B. bifurcata*, both ω3 and ω6, presented the same tendency, with greater production being verified in summer (3.91 ± 0.15 µg/mg of ω3 and 5.21 ± 0.16 µg/mg of ω6 (Table 1)) and lower in autumn (3.16 ± 0.05 µg/mg of ω3 and 4.20 ± 0.11 µg/mg of ω6 (Table 1)), reaching a 19% reduction in the production of both ω3 and ω6.

More significant ω3 contents were quantified in *U. lactuca* collected in winter (1.16 ± 0.02 µg/mg, Table 1) at concentrations 27–40% higher than the rest of the seasons. In terms of ω6, lower amounts were verified in winter (0.69 ± 0.02 µg/mg), autumn (0.83 ± 0.02 µg/mg), summer (0.95 ± 0.03 µg/mg), and spring (1.01 ± 0.01 µg/mg) (Table 1).

The h/H ratio depends on the values of the MUFA and PUFA and palmitic and myristic acids, and thus slight fluctuations were also seen during the seasons, especially for *F. spiralis* and *U. lactuca*. In the samples of *F. spiralis*, there was an increase from 2.54 (obtained in samples from spring and autumn) to 2.74 (in winter) and 2.78 (in summer) (Table 1). The same tendency was verified in the literature, with samples from S. Maria (Azores, Portugal) presenting an increase in the h/H ratio from 1.59 (in winter) to 1.89 (in summer) [12]. The opposite was verified for *U. lactuca*, in which the ratio increased from 1.17 (in summer) and 1.24 (in spring) to 1.39 (autumn) and 1.41 (winter) (Table 1). Regarding *B. bifurcata*, the increase was more subtle, increasing from 3.37 (in summer) and 3.39 (in autumn) to 3.45 (in both winter and spring) (Table 1).

Studies on the seasonal variations of the FA content in macroalgae, namely *Fucus spiralis* and *Ulva lactuca*, have concluded that the environmental conditions and life cycle associated with the seasons play an important role in the FA contents.

Paiva et al. [12] evaluated the seasonal variability (summer and winter) of *Fucus spiralis* collected from two islands of the Azores archipelago (S. Miguel and S. Maria). Overall, the samples from S. Maria presented higher lipid amounts in summer than the ones from S. Miguel, and this was attributed to the warmer water temperature. Higher contents of SFA were obtained from S. Maria and higher content of MUFA and PUFA were quantified from samples collected from S. Miguel in summer and winter, respectively [12]. S. Miguel island was also the collection location in a study that compared the composition of samples harvested in the juvenile (October) and mature (May) phase of the life cycle of *F. spiralis* [16]. Samples from the juvenile phase presented higher FA contents, in addition to presenting FA which were not identified in the mature phase, such as pentadecanoic acid, palmitoleic acid, and stearic acid [16]. In the present study, contrary to the previously mentioned studies, higher SFA, MUFA, and PUFA were quantified in summer and spring, and the FA mentioned were identified in all seasons.

**Figure 2:**
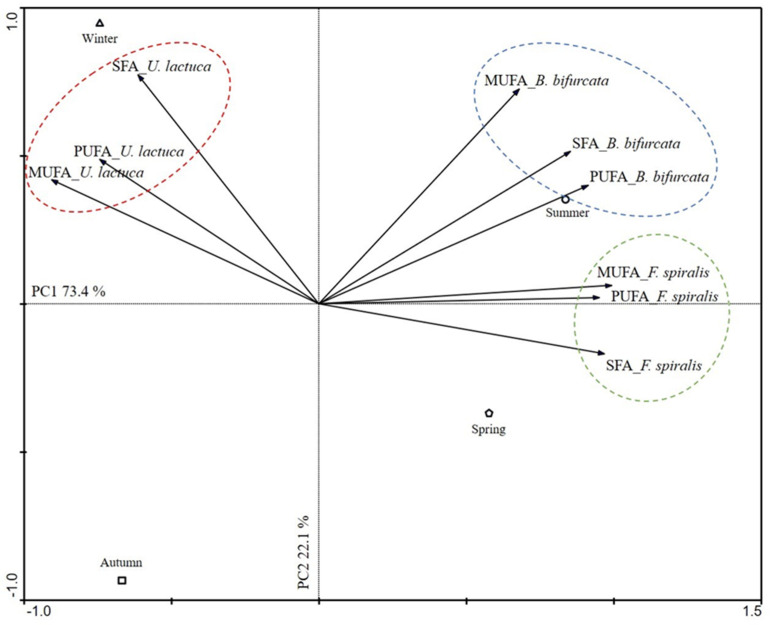
Biplot diagram from principal component analysis (PCA) relating the classes of FA (SFA, MUFA, and PUFA) present in *Ulva lactuca*, *Bifurcaria bifurcata,* and *Fucus spiralis* in different seasons (winter, spring, summer, and autumn).

With the analysis of main components, it was possible to corroborate the results previously described. Concretely, the first main plan (composed of components PC1 and PC2) explains 95.6% of the total variance of the data (Figure 2). PC1 was the most significant, explaining 73.4% of the variance (Figure 2).

The FA profile seemed to be influenced by the seasonal variations of all macroalgae studied. Thus, the results showed the existence of a correlation between the SFA, MUFA, and PUFA for each of the macroalgae under study (Figure 2). Therefore, the studied Ochrophyta species, *F. spiralis* and *B. bifurcata,* present a similar pattern when harvested in the summer, with *B. bifurcata* presenting greater FA contents in this season than *F. spiralis*. For *F. spiralis*, both summer and spring are beneficial for the production of FA. In winter and autumn, the productive behavior of these macroalgae is the opposite, with a decrease in the FA content (Figure 2).

As for *U. lactuca*, the behavior presented is opposite to that of the other species (*F. spiralis* and *B. bifurcata*), with higher FA production in winter followed by autumn. Summer and spring are the less beneficial seasons for the production of FA in *U. lactuca* (Figure 2).

These results suggest that the FA production in *B. bifurcata* and *F. spiralis* is stimulated by higher temperatures, while the contrary is seen in *U. lactuca*, with greater FA production at lower temperatures.

The effect of the seasons on the three brown macroalgae, *F. spiralis*, *B. bifurcata,* and *S. polyschides*, yielded distinct FA compositions and seasonal variabilities, suggesting that the FA profile could be linked to the species and not just taxonomic groups [36]. Nevertheless, this link could only be confirmed with further studies involving a larger number of algae species.

*Ulva lactuca* has been the focus of some articles that studied the seasonal variations of its biochemical components. Schmid et al. [36] compared the FA composition of samples harvested in June and November from Western Ireland, whereas Khairy and El-Shafay [43] and Mohy El-Din [44] evaluated the seasonal effects on the biochemical composition of samples from Egypt, first from spring to autumn and then throughout all seasons. Samples collected in Egypt and Ireland presented higher FA concentrations in spring and summer, respectively [36,43,44]. These results were contrary to those obtained in the present study, which found higher FA concentration in winter.

As previously mentioned, different results can be due to the environmental conditions caused by the distinct geographical locations, and thus the native environmental abiotic factors such as pH, light, water temperature, salinity, to which algae are exposed [36]. However, other factors could have also contributed to the differences between the results reported in this study and the ones reported in previous studies. Sampling, sample preparation (drying methods), and the quantification methodologies employed (and any modifications to the methods) in addition to the use of biomass vs. extracts for the FA analysis could have contributed to the dissimilar results [22,35]. The present study quantified the FA profile through direct transesterification of the freeze-dried biomass, while most of the previous studies first extracted the crude lipids and then proceeded to transesterification. A comparative study of the different sample preparations and the use of biomass vs. extract could be of interest to further understand their influence on the quantifications and if they play a part in the differences of the results.

The evaluation of the lipidic profile of different species of macroalgae, as well as its seasonal variations, is important to understand the mechanism employed in the production of its components but also to aid in the choice of harvesting time to obtain biomass with the highest amounts of the FA of interest. This is valuable knowledge to evaluate macroalgae’s potential not only as a natural nutritious food source, but also for potential applications in industry.

## 3. Materials and Methods

### 3.1. Reagents

A fatty acid methyl ester mix was used as GC standards (SupelcoTM 37 Component FAME Mix, Sigma-Aldrich). All solvents used for sample preparation were of analytical grade and the solvents used for GC analysis were of HPLC grade.

### 3.2. Sample Collection and Preparation

Macroalgae were collected in different seasons during 2018–2019, from various locations on the Peniche coast, Portugal. Samples of *Fucus spiralis* were collected from Marques Neves Beach (39°22′11.3′′ N, 9°23′09.9′′ W), *Ulva lactuca* from Óbidos Lagoon (39°25′01.6′′ N 9°12′50.1′′ W), and *Saccorhiza polyschides* and *Bifurcaria bifurcata* were collected at Baleal Beach (39°22′28.2′′ N 9°20′24.3′′ W and 39°37′68.32′′ N 9°34′01.12′′ W, respectively).

Due to low environmental sources, samples from *Saccorhiza polyschides* were only collected in the summer.

After collection, the macroalgae were transported in coolers from the harvest site to the lab, where they were screened and washed successively with seawater and distilled water. The samples were then freeze-dried (Labogene, CoolSafe 55-4) and ground.

### 3.3. Determination of the Fatty Acid Profile

The fatty acid profile was determined by acid-catalyzed direct transesterification following an adaptation of the methodology described by Fernández et al. [45].

Briefly, 50 mg of freeze-dried samples (3 replicates for each sample) were weighed and poured into test tubes, 2 mL of a methanol solution with 2% sulfuric acid was added, and the mixture was heated at 80 °C for 2 h. After cooling to room temperature, 1 mL of miliQ water and 2 mL of n-hexane were added, and the mixture was vortexed for 1 min and centrifuged at 1000 rpm for 5 min. Then, 1 mL of the upper organic phase was withdrawn and transferred to gas chromatography (GC) vials for analysis of the fatty acids’ methyl esters.

The samples were then injected into a gas chromatograph (Thermo Scientific, Finnigan-trace GC Ultra) equipped with a flame ionization detector (FID), an autosampler (AS 3000, Thermo Electron Corporation), and a TR-FAME capillary column (Thermo TR-FAME, 60 m × 0.25 mm ID × 0.25 µm film thickness). The injector (operating in splitless mode) and the detector temperatures were set at 250 and 280 °C, respectively. The column temperature was initially set at 75 °C for 1 min, then raised at 5 °C min^−1^ to 170 °C and held for 10 min followed by an increase at 5 °C min^−1^ to 190 °C and maintained for 10 min, and finally raised to 240 °C at 2 °C min^−1^ and held for 10 min. Helium was used as carrier gas at a flow rate of 1.5 mL min^−1^. Air and hydrogen were supplied to the detector at flow rates of 350 and 35 mL min^−1^, respectively.

The fatty acid profile was determined by comparing the resulting retention times with a 36-fatty acid standard (Supelco 37 component FAME Mix) and the results were expressed as µg of fatty acid/mg of dry macroalgae.

### 3.4. Data Analysis

All measurements were done in triplicate. In order to study the concentration, when comparing the seasons (spring, summer, autumn, and winter) samples were separated by each group of fatty acids (C4:0, C6:0, C8:0, C10:0, C12:0, C13:0, C14:0, C15:0, C16:0, C17:0, C18:0, C20:0, C21:0, C22:0, C23:0, C24:0, C14:1, C15:1, C16:1, C17:1, C18:1ω9t, C18:1ω9c, C18:1ω7, C20:1ω9, C24:1ω9, C22:1ω9, C18:3ω3, C18:4, C18:5ω3, C20:4ω3, C20:5ω3, C21:5ω3, C22:5ω3, C22:6ω3, C18:2ω6t, C18:2ω6c, C18:3ω6, C20:3ω6, C20:4ω6, C22:3ω6, C22:5ω6, C16:2ω4, C20:2 and C22:2) and for each species (*Bifurcaria bifurcata, Fucus spiralis*, *Saccorhiza polyschides,* and *Ulva lactuca*), an analysis of variance (ANOVA) with a factor was performed [46]. All requirements inherent to the performance of the ANOVA (namely, normality of data and homogeneity of variances) have been validated. Whenever these have not been fulfilled, the analysis was carried out using the non-parametric Kruskal–Wallis test [46]. To perform multiple comparisons, and whenever applicable, Tukey HSD multiple comparisons tests (for the cases studied using ANOVA) or Games-Howell (for the cases studied using the Kruskal–Wallis test) were used [46]. All results were considered statistically significant at the 5% level (that is, whenever *p*-value < 0.05). Whenever applicable, the results are presented as mean ± standard deviation (SD). Statistical analysis was performed using IBM SPSS Statistics 27 (Copyright IBM Corp. ©1989–2019, Armonk, NY 10504-1722, USA). A principal component analysis (PCA), based on correlation matrices, was performed in order to identify the main associations among the species (*Bifurcaria bifurcata, Fucus spiralis,* and *Ulva lactuca*) with the classes of FA produced (SFA, MUFA, and PUFA) and sampling periods (spring, summer, autumn, and winter) [47]. The principal component analysis (PC1 and PC2) provides information on the most meaningful parameters, which describe a whole dataset, affording data reduction with minimum loss of original information [48]. Although the results concerning the first two components were presented, the others were also analyzed. All calculations were performed with the CANOCO version 4.5 software (Copyright Petr Smilauer © 2012–2019, Ithaca, NY 14850, USA).

## 4. Conclusions

*Fucus spiralis*, *Bifurcaria bifurcata*, *Saccorhiza polyschides,* and *Ulva lactuca* showed potential to be used as sources of FA of interest for the human diet to supplement a healthy diet. The studied species were found to possess FA of interest for human nutrition, such as oleic acid, ARA, ALA, and LA. *Fucus spiralis* presented higher quantities of FA overall, *Bifurcaria bifurcata* presented higher concentrations of PUFA, and *Ulva lactuca* and *Saccorhiza polyschides* showed higher quantities of SFA. *U. lactuca* and *B. bifurcata* were the species that presented the “healthiest” ω6/ω3 ratio ratios, and all the species presented high h/H ratios. The FA profile was found to be influenced by the seasons. In Ochrophyta, the production of the FA overall seemed to be induced by spring and summer while in Chlorophyta the opposite seemed to happen, with higher production in winter. Individual FA were also influenced by the seasons with some of them reaching differences close to 40–50% between seasons, for instance in the case of the amount of oleic acid in *F. spiralis* or the amount of EPA in *B. bifurcata*. Some distinctions between published results and the results presented could be caused not only by the seasons, geographical harvest locations, or life cycle of the samples but also different quantification methodologies, such as sample preparation and direct vs. indirect transesterification, which can be a factor influencing the resulting FA profiles. Considering this, further studies should be performed to better understand these factors’ influence on the results.

## Figures and Tables

**Table 1:** Seasonal mean fatty acids composition of *Fucus spiralis*, *Bifurcaria bifurcata*, *Ulva lactuca,* and *Saccorhiza polyschides*, presented as µg of fatty acid/mg of macroalgae. For *Saccorhiza polyschides* only summer data are presented, due to lack of samples from the other seasons.

	*F. spiralis*				*B. bifurcata*				*U. lactuca*				*S. polyschides*
	Winter	Summer	Spring	Autumn	Winter	Summer	Spring	Autumn	Winter	Summer	Spring	Autumn	Summer
*C4:0*	0.58 ± 0.00	0.58 ± 0.02	0.57 ± 0.03	0.58 ± 0.03	0.57 ± 0.01	0.57 ± 0.01	0.58 ± 0.03	0.57 ± 0.02	0.63 ± 0.01 ^a^	0.87 ± 0.04 ^b^	0.60 ± 0.02 ^a^	0.65 ± 0.03 ^a^	0.80 ± 0.01
*C6:0*	0.53 ± 0.01	0.52 ± 0.01	0.52 ± 0.03	0.51 ± 0.01	0.72 ± 0.04	0.69 ± 0.02	0.70 ± 0.01	0.69 ± 0.00	0.60 ± 0.01	0.54 ± 0.01	0.58 ± 0.01	0.62 ± 0.01	0.51 ± 0.01
*C8:0*	nd	nd	nd	nd	nd	nd	nd	nd	0.51 ± 0.01 ^ac^	0.64 ± 0.02 ^b^	0.49 ± 0.00 ^a^	0.53 ± 0.01 ^bc^	0.63 ± 0.01
*C10:0*	nd	nd	nd	nd	nd	nd	nd	nd	0.73 ± 0.01 ^a^	0.55 ± 0.02 ^b^	0.54 ± 0.01 ^b^	0.67 ± 0.02 ^c^	nd
*C12:0*	0.53 ± 0.00	0.50 ± 0.00	0.52 ± 0.00	0.53 ± 0.01	nd	nd	nd	nd	0.70 ± 0.02 ^a^	0.54 ± 0.00 ^bc^	0.53 ± 0.00 ^b^	0.65 ± 0.03 ^ac^	0.53 ± 0.01
*C13:0*	nd	nd	nd	nd	nd	nd	nd	nd	0.46 ± 0.02 ^a^	0.31 ± 0.02 ^b^	0.31 ± 0.01 ^b^	0.45 ± 0.03 ^a^	0.31 ± 0.01
*C14:0*	2.74 ± 0.03	4.01 ± 0.25	3.87 ± 0.27	2.93 ± 0.47	0.96 ± 0.04 ^a^	1.06 ± 0.03 ^b^	0.97 ± 0.04 ^a^	0.93 ± 0.03 ^a^	0.66 ± 0.00 ^a^	0.64 ± 0.01 ^ab^	0.53 ± 0.01 ^c^	0.60 ± 0.03 ^bc^	0.89 ± 0.04
*C15:0*	0.30 ± 0.00 ^a^	0.33 ± 0.01 ^ab^	0.34 ± 0.01 ^b^	0.31 ± 0.02 ^a^	0.24 ± 0.01	0.25 ± 0.01	0.24 ± 0.00	0.22 ± 0.01	0.39 ± 0.01 ^c^	0.28 ± 0.01 ^d^	0.28 ± 0.00 ^d^	0.39 ± 0.01 ^c^	0.27 ± 0.01
*C16:0*	4.38 ± 0.05 ^a^	5.92 ± 0.27 ^ab^	6.41 ± 0.33 ^b^	4.66 ± 0.80 ^a^	3.50 ± 0.25 ^ce^	3.93 ± 0.21 ^d^	3.72 ± 0.25 ^de^	3.31 ± 0.14 ^c^	6.20 ± 0.32 ^f^	5.58 ± 0.33 ^g^	5.58 ± 0.30 ^g^	5.32 ± 0.32 ^g^	3.63 ± 0.19
*C17:0*	0.12 ± 0.00 ^a^	0.16 ± 0.00 ^b^	0.15 ± 0.01 ^b^	0.13 ± 0.00 ^a^	0.16 ± 0.01 ^cd^	0.18 ± 0.03 ^c^	0.15 ± 0.00 ^d^	0.15 ± 0.00 ^d^	nd	nd	nd	nd	0.12 ± 0.00
*C18:0*	0.56 ± 0.01 ^a^	0.95 ± 0.02 ^b^	0.79 ± 0.03 ^c^	0.59 ± 0.03 ^a^	0.48 ± 0.01	0.49 ± 0.01	0.48 ± 0.00	0.47 ± 0.02	0.49 ± 0.00	0.49 ± 0.01	0.48 ± 0.01	0.48 ± 0.01	0.51 ± 0.01
*C20:0*	1.97 ± 0.02	1.94 ± 0.09	2.31 ± 0.10	1.98 ± 0.34	nd	nd	nd	nd	nd	nd	nd	nd	0.87 ± 0.05
*C21:0*	nd	nd	nd	nd	nd	nd	nd	nd	0.22 ± 0.00	0.22 ± 0.00	0.22 ± 0.00	0.22 ± 0.00	0.21 ± 0.00
*C22:0*	nd	nd	nd	nd	0.69 ± 0.01	0.69 ± 0.04	0.69 ± 0.00	0.65 ± 0.05	0.91 ± 0.03 ^a^	0.83 ± 0.02 ^b^	0.86 ± 0.02 ^b^	0.86 ± 0.02 ^b^	nd
*C23:0*	nd	nd	nd	nd	0.20 ± 0.00	0.19 ± 0.01	0.20 ± 0.01	0.19 ± 0.01	nd	nd	nd	nd	nd
*C24:0*	0.46 ± 0.00 ^a^	0.53 ± 0.01 ^b^	0.50 ± 0.01 ^c^	0.47 ± 0.01 ^d^	0.48 ± 0.01	0.46 ± 0.02	0.46 ± 0.01	0.46 ± 0.02	0.44 ± 0.00 ^e^	0.42 ± 0.00 ^f^	0.42 ± 0.00 ^f^	0.43 ± 0.01 ^e^	nd
***ΣSFA***	12.16 ± 0.07	15.44 ± 0.38	15.99 ± 0.44	12.69 ± 0.99	8.00 ± 0.26	8.51 ± 0.22	8.18 ± 0.25	7.64 ± 0.16	12.94 ± 0.32	11.89 ± 0.33	11.41 ± 0.30	11.87 ± 0.33	9.28 ± 0.20
*C14:1*	0.24 ± 0.00 ^a^	0.26 ± 0.00 ^b^	0.25 ± 0.00 ^c^	0.24 ± 0.00 ^a^	0.32 ± 0.02	0.36 ± 0.04	0.33 ± 0.01	0.30 ± 0.00	0.33 ± 0.01 ^d^	0.25 ± 0.02 ^e^	0.26 ± 0.00 ^de^	0.32 ± 0.00 ^d^	0.26 ± 0.01
*C15:1*	0.30 ± 0.01 ^a^	0.27 ± 0.00 ^b^	0.29 ± 0.00 ^c^	0.29 ± 0.01 ^ac^	0.27 ± 0.00	0.27 ± 0.00	0.27 ± 0.00	0.27 ± 0.01	0.41 ± 0.00 ^g^	0.28 ± 0.00 ^h^	0.29 ± 0.00 ^gh^	0.35 ± 0.03 ^g^	0.28 ± 0.00
*C16:1*	0.69 ± 0.01 ^a^	0.81 ± 0.02 ^ab^	0.87 ± 0.04 ^b^	0.68 ± 0.09 ^a^	0.89 ± 0.05	0.85 ± 0.05	0.85 ± 0.05	0.83 ± 0.02	0.99 ± 0.03 ^a^	0.90 ± 0.07 ^ab^	0.65 ± 0.02 ^c^	0.87 ± 0.04 ^b^	0.91 ± 0.05
*C17:1*	0.25 ± 0.00 ^a^	0.28 ± 0.00 ^b^	0.28 ± 0.01 ^b^	0.25 ± 0.01 ^a^	0.22 ± 0.00 ^c^	0.25 ± 0.01 ^d^	0.23 ± 0.00 ^e^	0.21 ± 0.01 ^c^	0.24 ± 0.00 ^f^	0.22 ± 0.00 ^g^	0.23 ± 0.00 ^h^	0.24 ± 0.00 ^f^	nd
*C18:1ω9t*	0.22 ± 0.00 ^a^	0.24 ± 0.00 ^ab^	0.29 ± 0.01 ^b^	0.22 ± 0.00 ^a^	0.24 ± 0.00 ^c^	0.26 ± 0.01 ^d^	0.23 ± 0.00 ^c^	0.23 ± 0.01 ^c^	0.41 ± 0.01 ^e^	0.23 ± 0.00 ^f^	0.28 ± 0.00 ^g^	0.28 ± 0.00 ^g^	nd
*C18:1ω9c*	5.09 ± 0.05 ^a^	10.98 0.52 ^b^	8.71 0.75 ^c^	5.19 0.79 ^a^	2.33 0.17	2.40 0.14	2.27 0.18	2.22 0.10	0.53 ± 0.01 ^de^	0.78 ± 0.04 ^d^	0.81 ± 0.02 ^d^	0.51 ± 0.01 ^e^	1.79 0.16
*C18:1ω7*	0.24 ± 0.00 ^a^	0.26 ± 0.00 ^b^	0.28 ± 0.01 ^c^	0.23 ± 0.00 ^a^	0.28 ± 0.00	0.28 ± 0.01	0.31 ± 0.03	0.30 ± 0.01	2.49 0.12 ^d^	1.13 ± 0.08 ^e^	1.26 ± 0.03 ^e^	1.92 0.11 ^f^	0.57 ± 0.03
*C20:1ω9*	nd	nd	nd	nd	nd	nd	nd	nd	0.22 ± 0.00 ^a^	0.22 ± 0.01 ^b^	0.23 ± 0.00 ^c^	0.22 ± 0.00 ^d^	nd
*C24:1ω9*	0.58 ± 0.02	0.52 ± 0.02	0.56 ± 0.02	0.57 ± 0.05	0.30 ± 0.01	0.33 ± 0.05	0.30 ± 0.01	0.30 ± 0.01	0.25 ± 0.01 ^a^	0.22 ± 0.00 ^b^	0.25 ± 0.01 ^a^	0.27 ± 0.02 ^a^	nd
*C22:1ω9*	0.29 ± 0.00 ^ab^	0.28 ± 0.00 ^a^	0.31 ± 0.01 ^b^	0.27 ± 0.01 ^a^	0.41 ± 0.02 ^cd^	0.43 ± 0.01 ^d^	0.43 ± 0.01 ^c^	0.36 ± 0.01 ^e^	0.25 ± 0.00 ^f^	0.21 ± 0.00 ^g^	0.22 ± 0.00 ^h^	0.22 ± 0.00 ^i^	0.23 ± 0.01
***ΣMUFA***	7.90 ± 0.06	13.90 ± 0.52	11.84 ± 0.75	7.96 ± 0.79	5.26 ± 0.18	5.42 ± 0.17	5.23 ± 0.19	5.01 ± 0.10	6.12 ± 0.13	4.44 ± 0.11	4.48 ± 0.04	5.20 ± 0.13	4.03 ± 0.17
*C18:3ω3 (ALA)*	nd	nd	nd	nd	1.03 ± 0.07 ^a^	1.26 ± 0.10 ^b^	1.10 ± 0.07 ^c^	0.95 ± 0.05 ^ac^	0.74 ± 0.02 ^d^	0.34 ± 0.01 ^e^	0.43 ± 0.01 ^f^	0.47 ± 0.01 ^g^	nd
*C18:5ω3*	nd	nd	nd	nd	0.17 ± 0.00	0.17 ± 0.01	0.17 ± 0.00	0.17 ± 0.00	0.21 ± 0.01 ^a^	0.19 ± 0.00 ^b^	0.21 ± 0.00 ^a^	0.20 ± 0.01 ^a^	0.16 ± 0.00
*C20:4ω3*	0.85 ± 0.01	0.82 ± 0.01	0.88 ± 0.02	0.84 ± 0.03	0.74 ± 0.02	0.74 ± 0.05	0.72 ± 0.01	0.70 ± 0.02	nd	nd	nd	nd	nd
*C20:5ω3 (EPA)*	1.58 ± 0.01	1.58 ± 0.07	1.89 ± 0.09	1.40 ± 0.23	0.60 ± 0.02 ^a^	0.71 ± 0.02 ^b^	0.86 ± 0.05 ^c^	0.49 ± 0.01 ^d^	nd	nd	nd	nd	0.41 ± 0.02
*C21:5ω3*	nd	nd	nd	nd	0.38 ± 0.01	0.39 ± 0.07	0.37 ± 0.02	0.34 ± 0.01	nd	nd	nd	nd	nd
*C22:5ω3* *(DPA)*	nd	nd	nd	nd	0.39 ± 0.02	0.46 ± 0.07	0.40 ± 0.01	0.36 ± 0.01	0.22 ± 0.00 ^a^	0.16 ± 0.00 ^b^	0.17 ± 0.00 ^c^	0.18 ± 0.00 ^d^	nd
*C22:6ω3 (DHA)*	nd	nd	nd	nd	0.17 ± 0.01	0.18 ± 0.01	0.17 ± 0.00	0.16 ± 0.01	nd	nd	nd	nd	nd
***Σ ω3***	2.43 ± 0.02	2.40 ± 0.07	2.77 ± 0.09	2.24 ± 0.23	3.49 ± 0.08	3.91 ± 0.15	3.79 ± 0.09	3.16 ± 0.05	1.16 ± 0.02	0.69 ± 0.01	0.81 ± 0.01	0.85 ± 0.02	0.57 ± 0.02
*C18:2ω6t* *(LA)*	nd	0.24 ± 0.00	nd	nd	nd	nd	nd	nd	nd	nd	nd	nd	nd
*C18:2ω6c (LA)*	1.74 ± 0.00 ^a^	2.19 ± 0.10 ^ab^	2.46 ± 0.15 ^b^	1.71 ± 0.29 ^a^	0.51 ± 0.02 ^c^	0.68 ± 0.03 ^d^	0.61 ± 0.03 ^e^	0.50 ± 0.02 ^c^	0.46 ± 0.02 ^f^	0.49 ± 0.02 ^f^	0.56 ± 0.01 ^g^	0.38 ± 0.01 ^h^	0.61 ± 0.05
*C18:3ω6 (GLA)*	0.31 ± 0.00 ^a^	0.36 ± 0.01 ^b^	0.35 ± 0.01 ^b^	0.30 ± 0.01 ^a^	0.26 ± 0.01	0.29 ± 0.03	0.25 ± 0.01	0.26 ± 0.01	0.22 ± 0.00 ^c^	0.22 ± 0.00 ^c^	0.23 ± 0.00 ^d^	0.22 ± 0.00 ^e^	0.24 ± 0.01
*C20:3ω6*	0.34 ± 0.00 ^a^	0.45 ± 0.01 ^b^	0.45 ± 0.02 ^b^	0.34 ± 0.02 ^a^	0.45 ± 0.01 ^cd^	0.47 ± 0.01 ^c^	0.47 ± 0.02 ^c^	0.43 ± 0.02 ^d^	nd	nd	nd	nd	0.26 ± 0.01
*C20:4ω6 (AA)*	4.21 ± 0.04 ^a^	5.94 ± 0.28 ^b^	5.61 ± 0.35 ^ab^	4.22 ± 0.61 ^a^	2.63 ± 0.15 ^c^	3.10 ± 0.15 ^d^	2.94 ± 0.15 ^d^	2.45 ± 0.11 ^c^	nd	nd	nd	nd	2.07 ± 0.19
*C22:3ω6*	nd	nd	nd	nd	0.33 ± 0.02 ^ab^	0.36 ± 0.02 ^c^	0.35 ± 0.01 ^ac^	0.31 ± 0.01 ^b^	nd	0.24 ± 0.01 ^a^	0.22 ± 0.00 ^b^	0.23 ± 0.01 ^ab^	nd
*C22:5ω6*	nd	nd	nd	nd	0.27 ± 0.02	0.30 ± 0.03	0.28 ± 0.01	0.25 ± 0.00	nd	nd	nd	nd	nd
***Σ ω6***	6.60 ± 0.04	9.18 ± 0.30	8.87 ± 0.38	6.56 ± 0.68	4.45 ± 0.15	5.21 ± 0.16	4.91 ± 0.16	4.20 ± 0.11	0.69 ± 0.02	0.95 ± 0.03	1.01 ± 0.01	0.83 ± 0.02	3.18 ± 0.20
*C16:2ω4*	0.27 ± 0.01 ^a^	0.25 ± 0.00 ^b^	0.27 ± 0.00 ^a^	0.28 ± 0.01 ^c^	0.25 ± 0.00	0.25 ± 0.01	0.26 ± 0.00	0.25 ± 0.01	0.46 ± 0.01 ^d^	0.29 ± 0.00 ^e^	0.30 ± 0.00 ^e^	0.41 ± 0.03 ^de^	0.27 ± 0.01
C18:4 *	1.96 ± 0.01	1.53 ± 0.04	2.00 ± 0.07	1.88 ± 0.26	1.32 ± 0.06 ^a^	1.37 ± 0.06 ^a^	1.38 ± 0.04 ^a^	1.17 ± 0.03 ^b^	1.04 ± 0.02 ^c^	0.66 ± 0.01 ^d^	0.73 ± 0.00 ^e^	0.75 ± 0.01 ^e^	1.21 ± 0.04
*C20:2*	0.34 ± 0.00 ^a^	0.35 ± 0.01 ^ab^	0.42 ± 0.02 ^b^	0.32 ± 0.02 ^a^	0.36 ± 0.01	0.35 ± 0.02	0.34 ± 0.02	0.34 ± 0.01	0.23 ± 0.00	0.22 ± 0.00	0.22 ± 0.01	0.22 ± 0.00	nd
*C22:2*	nd	nd	nd	nd	0.23 ± 0.01 ^a^	0.28 ± 0.00 ^b^	0.24 ± 0.00 ^abc^	0.23 ± 0.00 ^c^	nd	nd	nd	nd	nd
***ΣPUFA***	11.59 ± 0.27	13.71 ± 0.40	14.32 ± 0.48	11.28 ± 0.81	10.11 ± 0.18	11.36 ± 0.23	10.92 ± 0.19	9.35 ± 0.13	3.57 ± 0.04	2.82 ± 0.04	3.07 ± 0.02	3.06 ± 0.03	5.23 ± 0.20
***PUFA/*** ***SFA***	0.95 ± 0.05	0.89 ± 0.12	0.90 ± 0.17	0.89 ± 0.43	1.26 ± 0.27	1.34 ± 0.50	1.33 ± 0.26	1.22 ± 0.23	0.28 ± 0.03	0.24 ± 0.03	0.27 ± 0.03	0.26 ± 0.04	0.60 ± 0.11
***FA***	31.65 ± 0.28	43.05 ± 0.75	42.15 ± 0.99	31.92 ± 1.51	23.37 ± 0.36	25.29 ± 0.36	24.33 ± 0.37	22.00 ± 0.23	22.63 ± 0.35	19.15 ± 0.35	18.96 ± 0.30	20.13 ± 0.35	18.54 ± 0.33
*** ω6*** *** ω3***	2.72 ± 0.06	3.83 ± 0.34	3.21 ± 0.37	2.93 ± 0.85	1.28 ± 0.21	1.33 ± 0.41	1.30 ± 0.19	1.33 ± 0.17	0.59 ± 0.05	1.38 ± 0.09	1.25 ± 0.04	0.98 ± 0.06	5.60 ± 0.80
***h/H***	2.74	2.78	2.54	2.54	3.45	3.37	3.45	3.39	1.41	1.17	1.24	1.39	2.05
***DHA/*** ***EPA***	nd	nd	nd	nd	0.28 ± 0.01	0.25 ± 0.01	0.19 ± 0.01	0.32 ± 0.02	nd	nd	nd	nd	nd

SFAs, saturated fatty acids; MUFAs, monounsaturated fatty acids; PUFAs, polyunsaturated fatty acids; ALA, α-linolenic acid; GLA, γ-linolenic acid; EPA, eicosapentaenoic acid; DPA, docosapentaenoic acid; DHA, docosahexaenoic acid; LA, linoleic acid; AA, arachidonic acid. “nd”, not detected. h/H, hypocholesterolemic (MUFA + PUFA)/hypercholesterolemic (C14:0 + C16:0) ratio. Values expressed as mean ± SD (n = 3). Means in the same row with unlike letters differ significantly (ANOVA, Tukey HSD, *p*-value < 0.05 or Kruskal–Wallis, Games-Howell, *p*-value < 0.05). * Contains C18:4ω1 and C18:4ω3.

## Data Availability

The data presented in this study are available on request from the corresponding author.

## Sample Availability

Samples of the compounds are not available from the authors.
